# A New NF-κB Inhibitor, MEDS-23, Reduces the Severity of Adverse Post-Ischemic Stroke Outcomes in Rats

**DOI:** 10.3390/brainsci12010035

**Published:** 2021-12-28

**Authors:** Elina Rubin, Agnese C. Pippione, Matthew Boyko, Giacomo Einaudi, Stefano Sainas, Massimo Collino, Carlo Cifani, Marco L. Lolli, Naim Abu-Freha, Jacob Kaplanski, Donatella Boschi, Abed N. Azab

**Affiliations:** 1 Department of Clinical Biochemistry and Pharmacology, Ben-Gurion University of the Negev, P.O. Box 653, Beer-Sheva 8410501, Israel; Elina.Rubin@perrigo.co.il (E.R.); yacovk2@gmail.com (J.K.); 2 Department of Drug Science and Technology, University of Turin, 10125 Turin, Italy; agnesechiara.pippione@unito.it (A.C.P.); stefano.sainas@unito.it (S.S.); marco.lolli@unito.it (M.L.L.); donatella.boschi@unito.it (D.B.); 3 Department of Anesthesiology and Critical Care, Soroka University Medical Center, Ben-Gurion University of the Negev, Beer-Sheva 8410501, Israel; matewboyko@gmail.com; 4 Pharmacology Unit, School of Pharmacy, University of Camerino, 62032 Camerino, Italy; giacomo.einaudi@unicam.it (G.E.); carlo.cifani@unicam.it (C.C.); 5 Department of Neuroscience “Rita Levi Montalcini”, University of Turin, 10125 Turin, Italy; massimo.collino@unito.it; 6 Institute of Gastroenterology and Hepatology, Soroka University Medical Center, Ben-Gurion University of the Negev, Beer-Sheva 8410501, Israel; abufreha@yahoo.de; 7 Department of Nursing, Ben-Gurion University of the Negev, P.O. Box 653, Beer-Sheva 8410501, Israel

**Keywords:** stroke, NF-κB, inflammation, ischemic stroke, depression, mortality

## Abstract

Aim: Nuclear factor kappa B (NF-κB) is known to play an important role in the inflammatory process which takes place after ischemic stroke. The major objective of the present study was to examine the effects of MEDS-23, a potent inhibitor of NF-κB, on clinical outcomes and brain inflammatory markers in post-ischemic stroke rats. Main methods: Initially, a Toxicity Experiment was performed to determine the appropriate dose of MEDS-23 for use in animals, as MEDS-23 was analyzed in vivo for the first time. We used the middle cerebral artery occlusion (MCAO) model for inducing ischemic stroke in rats. The effects of MEDS-23 (at 10 mg/kg, ip) on post-stroke outcomes (brain inflammation, fever, neurological deficits, mortality, and depression- and anxiety-like behaviours) was tested in several efficacy experiments. Key findings: MEDS-23 was found to be safe and significantly reduced the severity of some adverse post-stroke outcomes such as fever and neurological deficits. Moreover, MEDS-23 significantly decreased prostaglandin E2 levels in the hypothalamus and hippocampus of post-stroke rats, but did not prominently alter the levels of interleukin-6 and tumor necrosis factor-α. Significance: These results suggest that NF-κB inhibition is a potential therapeutic strategy for the treatment of ischemic stroke.

## 1. Introduction

Stroke is the second leading cause of death worldwide, causing 5.5 million deaths in 2016. More than half of stroke survivors are left with a disability and a third are dependent on others for the tasks of daily living [1].

Ischemic stroke is the most prevalent type of stroke among adults and accounts for more than 80% of all stroke cases [2]. The primary underlying cause of ischemic stroke is cerebral infarction (for example, due to embolism or atherosclerotic stenosis) [3]. Hypoxia and hypoglycemia start the ischemic cascade that leads to acute cell death (necrosis) and delayed programmed cell death (apoptosis). Despite the use of acute therapeutic interventions aimed at rapid reperfusion of tissue surrounding the ischemic core, various types of deficits originating from stroke necessitate long-term rehabilitation processes [3,4,5]. Of note, hematologic disorders such as coagulopathies and hematologic cell disorders are a commonly unrecognized cause of ischemic stroke in humans, necessitating different treatment approaches according to the particular underlying disorder [6]. Inflammation is a key contributor to the pathophysiology of ischemic stroke, playing a role in both secondary brain damage and the post-stroke repair process [7]. Post-ischemic stroke brain showed over-activation of nuclear factor (NF)-κB a transcription factor ubiquitously involved in the regulation of inflammation, cell proliferation and other cellular processes, where it seemingly contributed to the resulting tissue damage [8,9]. Thus, inhibition of NF-κB may reduce the post-stroke inflammatory reaction and mitigate the tissue damage and neurological deficits [10]. Consistent with this assumption, small molecules able to reduce NF-κB activation have been tested in rodent models of ischemic stroke producing desirable outcomes (e.g., suppressing production of inflammatory cytokines, reducing cerebral edema, infarct sizes, and neurological deficits) [10]. However, these compounds were not selective for NF-κB inhibition, and thus their ability to mitigate the adverse outcomes of ischemic stroke cannot be attributed (solely) to inhibition of NF-κB because they influence other cellular pathways.

The NF-κB pathway can be regulated by exogenous molecules at many levels, and thus several compounds have been developed for inhibition of its activation. Pippione et al. [11] recently discovered a new molecule (herein after referred to as MEDS-23) capable of specifically inhibiting the canonical NF-κB cascade at a nanomolar concentration (IC_50_ = 143 nM). Starting from the chemical IMD-0354 (Figure S1), a well-known NF-κB inhibitor [12,13,14], they used an acidic hydroxylated azole to mimic the electron withdrawing substituted phenol. This technique, called bioisosteric scaffold hopping, allows for obtaining compounds with modulated acidity and lipophilicity, hydrogen bond donor/acceptor capability, orientation of substituents and, consequently, can improve interaction with the target and/or in-vivo pharmacokinetic parameters [15,16,17]. Pippione et al. [11,18] reported that both MEDS-23 and IMD-0354 were inactive against isolated IKKβ enzymes but were both able to block the degradation of IκBα after inflammatory stimulus (TNFα) in Jurkat cells.

Taking into account the involvement of NF-κB in the pathophysiology of ischemic stroke, it has been suggested that NF-κB may be proven an important therapeutic target for post-stroke therapy [10,19,20]. We hypothesized that chronic treatment with the new NF-κB inhibitor MEDS-23 would improve clinical outcomes and reduce levels of brain inflammatory mediators in post-stroke rats.

Since MEDS-23 had never been tested in-vivo, we initially conducted a preliminary Toxicity Experiment in control (non-stroke) rats to determine the appropriate dose of MEDS-23 to be-used in the Efficacy Experiments in post-stroke rats. We evaluated the effects of MEDS-23 on adverse outcomes in post-ischemic stroke rats using the middle cerebral artery occlusion (MCAO) model. Moreover, we determined the effects of MEDS-23 on levels of the inflammatory mediators IL-6, TNF-α and PGE2 in various brain regions of post-stroke rats.

## 2. Materials and Methods

### 2.1. Animals

Male Sprague-Dawley rats with an initial weight of ~300 g were used throughout the studies. Rats were housed three per cage in the “Animal House” in Ben-Gurion University of the Negev (Israel) and maintained under controlled environmental conditions with ambient temperature 22 ± 1 °C, a relative humidity between 55–58%, and a photoperiod cycle of 12 h of light and 12 h dark. Rats were fed Purina Lab chow and water ad libitum (unless otherwise indicated). Rats were randomly allocated to the treatment groups of the study. Only rats without evident pathology such as signs of sickness, fever and abnormal social behavior were included in the study. Rats were maintained and treated according to the guidelines of the Committee for the Use and Care of Laboratory Animals in Ben-Gurion University of the Negev (Authorization ^#^ IL- 58-08-2019[E]). A few days before each experiment, rats were accustomed to the operators’ touch. This practice was performed to assure that during the actual experiment, rats would not have abrupt responses to the operators’ touch during measurement of body temperature (BT) and injections.

### 2.2. Measurement of Body Weight (BW)

BW was measured using a digital scale. At the start of each experiment, the average BW of all treatment groups was identical. During the course of the experiment, BW was measured daily in order to adjust the dose of MEDS-23 according to BW. In this context, post-stroke rats usually have a significant reduction in BW especially during the initial first few days after the surgical procedure.

### 2.3. Measurement of BT

BT was measured using a plastic-coated thermocouple probe (HL 600 Thermometer, Anristu Meter Co., Tokyo, Japan) inserted into the rectum. Rats were acclimated to the procedure two to three days before the initiation of the “official” experiments. BT was measured before the surgical procedure (baseline) and on the first, second and third day after the operation.

### 2.4. Surgical Procedures

The middle cerebral artery occlusion (MCAO) and sham operations were performed exactly as described previously [21,22]. Briefly, rats were anesthetized with ketamine 75 mg/kg and midazolam 3 mg/kg, both given intraperitoneally (i.p.). Anesthetized rats were subjected to either an MCAO surgery (which lasted 25–30 min) or a sham-operated surgery (which lasted 10–15 min) during which they could breathe spontaneously. As described previously [23], these surgical procedures are not associated with negative alterations in blood oxygenation (hypoxia) and pH, blood glucose and electrolyte levels, blood pressure (hypotension) or BT (hypothermia). At the end of the surgical procedures, all operated rats were injected (i.p.) with 10 mL of 0.9% saline solution to assure better recovery and make up for a lower water intake during the first day post-surgery. Of note, the use of anesthetics and the complexity of the surgical MCAO procedures can cause unexpected intra-operative death in particularly susceptible animals (the intra-operative death rate in the present study was approximately 7%).

### 2.5. Drug Treatment

The relatively large amounts of MEDS-23 needed for the conduction of the in-vivo experiments of the present study necessitated the use of a new synthetic scheme different from the one used in the originally developed protocol [11,24]. The authors took advantage of the recent knowledge acquired from their studies about hydroxylated azoles to improve synthesis and characterization of MEDS-23 [25,26] (for details, see Supplementary Material Section S1). MEDS-23 was dissolved in dimethyl sulfoxide (DMSO) in injection volumes that ranged between 0.1–0.2 mL according to animals’ BW. In the Toxicity Experiment, MEDS-23 was administered (i.p.) at a dose range between 1–100 mg/kg, and in the efficacy experiments it was given at a dose of 10 mg/kg.

### 2.6. Assessment of Neurological Score (NS)

The surgical operation for induction of stroke (MCAO) is a delicate and complex procedure which is not assured to be successful. Therefore, to assess the correctness of the MCAO and sham-operation procedures, each operated rat was monitored for post-surgical neurological deficits (NDs) as described below, in order to assign them to the appropriate group. The first assessment was performed two to three hours after the surgical procedure and upon full awakening from anesthesia. Thereafter, rats were tested once a day for another three days. An experienced observer who was blind to the type of surgical procedure examined each animal for visible NDs and scored them exactly as described previously [21,22]. Each animal was assigned an NS between zero and four. Rats in the MCAO group needed to have a NS ≥ 3 in order to be included in this group. In contrast, rats in the sham group were excluded if they presented any visible ND (NS > 0).

### 2.7. Assessment of Mortality and General Health Condition

Assessments of death/survival and general health condition were performed several times a day throughout the whole duration of the experiments. Assessment of general health condition included evaluation of the following features: general locomotion, social interaction with other rats, presence of diarrhea, presence of strong smell of feces or urine, significant changes in BT, significant changes in BW, external bleeding, and piloerection.

### 2.8. Behavioral Tests

Various models are used to test behavioral phenotypes in animals [27,28,29,30,31], however, they do not fulfill all recognized validity criteria. Due to the paucity of established validated animal models for mental disorders, this study utilized some of the most accepted and widely used models in translational psychiatry research [27,28,29,30,31] that are relevant to the present study. Behavioral studies were conducted during the light phase of the light/dark cycle. Before initiation of the behavioral tests, rats were acclimatized to housing conditions for several days and then subjected to the behavioral experiments.

#### 2.8.1. Open Field Test (OFT)

An open field test assesses the spontaneous activity of animals and serves as a control measure for other behavioral tests and a possible indicator of depressive- and manic-like behavior. The open field arena is made of a black box (60 cm [W] × 80 cm [L] × 60 cm [H]). Initially, rats were placed in the corner of the arena and monitored for 10 min. Sessions were videotaped by a camera placed approximately one meter above the center of the arena and subsequently assessed using a video-tracking system (Ethovision, Noldus, Wageningen, Netherlands). A 5% alcohol in water solution was used to clean the apparatus prior to introduction of each animal. The parameters that were analyzed are total distance travelled and mean velocity of movement.

#### 2.8.2. Sucrose Consumption Test (SCT)

This test is used to assess anhedonia—a behavioral feature of depression. The test was conducted as described previously [29,30] in order to assess the antidepressant-like effect of MEDS-23 in post-MCAO rats. The test was performed twice, once before conduction of the surgical procedures (baseline) and a second time at the end of the experiment after completion of the MEDS-23 treatment protocol. Sucrose 1% solution was prepared by dissolving 100 g of sucrose in 10 L of tap water. One day before the first test, rats were exposed to the sugary solution for one hour. On the experiment day, two different bottles, one containing regular water and one containing the sucrose solution were weighed using a digital scale. The pre-weighed bottles were put in each cage for 24 h, at the end of which they were weighed again. Sucrose consumption was calculated as grams of sucrose consumed per kilograms of BW using the following formula:

$$Sucrose\;consumption=\frac{\frac{weight\;of\;pre\;-\;weighted\;bottle\;-\;weight\;of\;post\;-\;session\;bottle\;}{100}}{\frac{total\;body\;weight\;of\;rats\;\;\left(g\right)\;in\;cage}{1000}}$$

#### 2.8.3. Elevated Plus-Maze Test (EPMT)

This test is used for the assessment of anxiety-like behavior (associated with depression) and risk-taking behavior (associated with mania). Rats were placed for five minutes in an elevated plus-maze consisting of two open arms (50 × 10 cm) and two walled arms (50 × 10 × 40 cm) with an open roof, arranged such that the two open arms were opposite to each other. The maze was elevated to a height of 50 cm. Rats were placed in the center of the maze facing one of the open arms. Sessions were videotaped by a camera placed two meters above the center of the maze and subsequently evaluated by two experienced observers who were blind to the specific treatment of each rat. The evaluated measures were: time spent in the open/walled arms and the number of entries to the open arm (defined as the entry of all four limbs into the arm) [27,28,29,30,31].

### 2.9. Blood and Tissue Collection and Preparation of Brain Homogenates

At the end of each experiment, surviving rats were anesthetized briefly with a mixture of 4% isoflurane in 100% oxygen and immediately euthanized by decapitation. Thereafter, blood, brains, stomach and kidney were collected simultaneously for further analysis. A volume of 6–8 mL blood was withdrawn from each rat into Heparin-containing tubes. Blood was then centrifuged at 3500 rpm for 10 min at 4 °C, and plasma was separated and kept at −80 °C. Brains were quickly extracted and washed in ice-cold 0.9% saline. The rontal cortex (FC), hypothalamus (HT) and hippocampus (HC) were gently excised on ice, cleaned and immediately transferred to −80 °C [21,22]. Subsequently, each sample was weighed and manually homogenized for 10 s in 500 μL of a cold phosphate-buffered saline solution containing protease/phosphatase inhibitors. Tissue homogenates were centrifuged at 10,000 rpm for 10 min at 4 °C. Supernatants were collected and immediately transferred to −80 °C. In the toxicity experiment, the stomach and kidney were extracted to evaluate adverse effects of MEDS-23 on these organs.

### 2.10. Determination of Renal, Hematological and Gastric Parameters

Blood levels of creatinine and urea were detected in the Biochemistry laboratory in Soroka University Medical Center in Beer-Sheva, Israel (hereafter, SUMC). Kidneys were weighed and structure was assessed for visible macroscopic structural changes. Moreover, hematological parameters including red blood cells (RBCs), white blood cells (WBCs), platelets (PLTs) and hemoglobin (Hb) were detected in the Hematology laboratory in SUMC. These parameters were tested to assess the effects of MEDS-23 on basic bone marrow function and the possibility of blood loss (for example, due to gastric ulcers). The integrity of the gastric mucosa and the presence of gastritis and/or ulcers were evaluated by a gastroenterologist (N.A-F.) who was blind to the treatment of each rat.

### 2.11. Measurement of Cytokines and PGE2 Levels

IL-6, PGE2 and TNF-α levels were determined by designated ELISA kits according to manufacturer’s protocol (R&D System, Minneapolis, MI, USA) as described previously [21,22].

### 2.12. Statistical Analysis and Presentation of the Data

Normally distributed data and continuous variables are presented as mean ± SEM. For survival estimation, a Log Rank (Mantel cox) test was used for comparison of the different groups. For evaluation of changes in different biochemical measurements, an independent-samples T test was used for between-group comparisons. A two-way ANOVA test (two-stage linear step-up procedure of Benjamini, Krieger and Yekutieli) was used for determining inter-group differences in brain cytokine levels. In all statistical analyses, values of *p* < 0.05 were considered statistically significant.

## 3. Results

### 3.1. Toxicity Experiment

For this experiment, we used 36 male rats divided to into six groups (six rats per group), as follows: (1) control—treated with vehicle (0.2 mL DMSO), (2) MEDS-23 1 mg/kg—treated with MEDS-23 1 mg/kg (dissolved in 0.2 mL DMSO), (3) MEDS-23 5 mg/kg—treated with MEDS-23 5 mg/kg, (4) MEDS-23 10 mg/kg—treated with MEDS-23 10 mg/kg, (5) MEDS-23 50 mg/kg—treated with MEDS-23 50 mg/kg, and, (6) MEDS-23 100 mg/kg—treated with MEDS-23 100 mg/kg. Treatment compounds were administered once daily (everyday between 08:00–10:00 a.m.) through i.p. injection for seven days. Treatment dose was adjusted to rats’ BW, which was measured daily before drug administration. The following parameters were monitored during the Toxicity Experiment: (1) BW, (2) mortality, (3) renal function parameters, (4) hematological parameters, and, (5) gastric mucosal integrity. In addition to these quantitative parameters, we also assessed qualitative parameters of “general well-being” as described in Methods (Section 2.7). The detailed results of the Toxicity Experiment can be seen in the supplementary files (Figure S2, and Tables S1–S4). Briefly, this experiment revealed that the administration of MEDS-23 at 100 mg/kg was extremely toxic and killed all the animals, while 50 mg/kg was associated with minor toxic effects. On the other hand, 1, 5 and 10 mg/kg did not cause any adverse effects in all tested parameters. Therefore, we chose to use the 10 mg/kg dose in the Efficacy Experiments.

### 3.2. Efficacy Experiments

We conducted four independent experiments aiming to examine the efficacy of MEDS-23 treatment in post-stroke rats. The combined number of rats in each treatment group throughout the experiments is shown in Table S5.

In all *Efficacy Experiments*, sham-operated and MCAO (stroke)-operated rats were treated with vehicle or MEDS-23 for a total of 15 days during which they were followed and assessed for evaluation of different study outcomes (e.g., BT, BW, NS and mortality). The first injection of vehicle/MEDS-23 was given at 4 h after the surgical procedure on day “zero”, to be followed by 14 single daily injections during the follow-up period. At the end of the follow-up period, animals were euthanized by decapitation and their brains were ousted for further determination of the levels of inflammatory mediators.

#### 3.2.1. Effects of MEDS-23 Treatment on BW of Post-Stroke Rats

**Figure 1:**
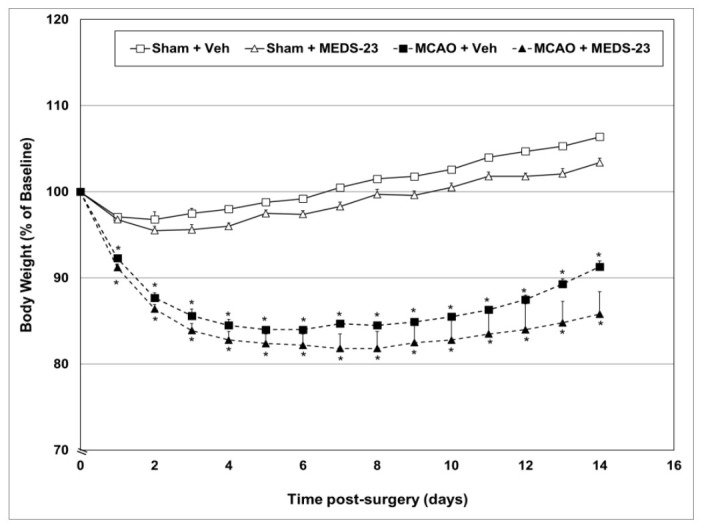
Effects of MEDS-23 treatment on BW of post-stroke rats. Sham- and MCAO-operated rats were treated with vehicle (DMSO 0.1 mL/rat) or MEDS-23 10 mg/kg for a total of 15 days through a single daily injection (i.p.). BW was measured every day before treatment. The figure represents the combined results of all four *Efficacy Experiments*. Data are presented as percentage of baseline BW, which is expressed as 100%. Each point represents mean ± SEM of a different number of rats in each group at the various time-points of the experiment, as shown in Table S5. Using dependent samples *t*-test—* *p* < 0.05 vs. Sham + Vehicle—at the same time point. MCAO—middle cerebral artery occlusion; Veh—vehicle.

Figure 1 presents the changes in rats’ BW during the experimental days. At baseline, the average BW did not differ between all treatment groups (mean ± SEM = 355.2 ± 3.1 g). As seen throughout the follow-up period, MCAO-operated rats had a significantly lower BW than those who underwent sham-surgery. MEDS-23 treatment was associated with a slight, non-significant decrease in BW in both sham- and MCAO-operated rats (Figure 1).

#### 3.2.2. Effects of MEDS-23 Treatment on BT of Post-Stroke Rats

**Figure 2:**
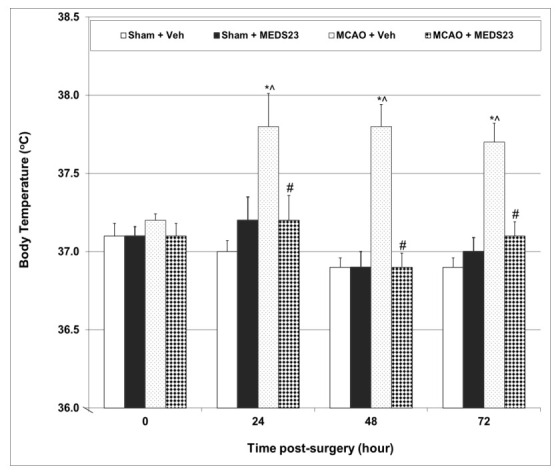
Effects of MEDS-23 treatment on BT of post-stroke rats. Sham- and MCAO-operated rats were treated with vehicle (DMSO 0.1 mL/rat) or MEDS-23 10 mg/kg for a total of 15 days through a single daily injection (i.p.). BT was measured before and at 24, 48 and 72 h post-surgery. The figure represents the results of a single efficacy experiment (very similar results were obtained in other experiments). Each point represents mean ± SEM of a different number of rats in each group at the various time-points of the experiment. At baseline, the sample size in each group was as follows: Sham + Vehicle = 19, Sham + MEDS-23 = 19, MCAO + Vehicle = 30, MCAO + MEDS-23 = 31. Using two-way ANOVA—* *p* < 0.05 vs. Sham + Vehicle, ^ *p* < 0.05 vs. Sham + MEDS-23, # *p* < 0.05 vs. MCAO + Vehicle—at the same time-point. MCAO—middle cerebral artery occlusion; Veh—vehicle.

Rats’ BT in all groups was measured simultaneously in a time-adjusted manner to avoid time-associated effects. Figure 2 presents the changes in BT in post-stroke rats. At baseline the average BT did not significantly differ between the groups. Subsequently, BT did not significantly differ between sham-operated rats irrespective of the treatment given (vehicle or MEDS-23). On the other hand, BT was significantly higher in vehicle-treated post-MCAO rats at these time-points. Treatment with MEDS-23 significantly attenuated the elevated BT in MCAO-operated rats at the same time-points (Figure 2).

#### 3.2.3. Effects of MEDS-23 Treatment on Levels of Brain Inflammatory Mediators in Post-Stroke Rats

**Figure 3:**
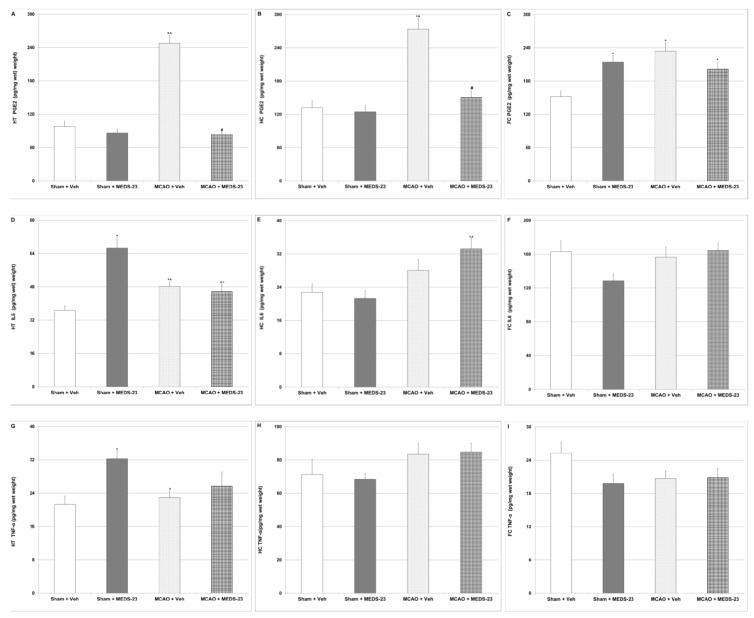
Effects of MEDS-23 treatment on brain inflammatory mediator levels in post-stroke rats. Sham- and MCAO-operated rats were treated with vehicle (DMSO 0.1 mL/rat) or MEDS-23 10 mg/kg for a total of 15 days through a single daily injection (i.p.). Brain regions were extracted as described in “Materials and Methods” at 14 days after surgery. PGE2 (**A**–**C**), IL-6 (**D**–**F**) and TNF-α (**G**–**I**) levels in HT (**A**,**D**,**G**), HC (**B**,**E**,**H**) and FC (**C**,**F**,**I**) were determined by specific ELISA kits. Each column is the mean ± SEM of 10 to 21 samples per group. Using two-way ANOVA test (two-stage linear step-up procedure of Benjamini, Krieger and Yekutieli)—* *p* < 0.05 vs. Sham + Veh; ^ *p* < 0.05 vs. Sham + MEDS-23; # *p* < 0.05 vs. MCAO + Veh—between-group comparisons. FC—frontal cortex, HC—hippocampus, HT—hypothalamus, IL-6—interleukin-6, MCAO—middle cerebral artery occlusion, PGE2—prostaglandin E2, TNF—tumor necrosis factor, Veh—vehicle.

We tested the effects of MEDS-23 on levels of IL-6, PGE2 and TNF-α in the brains of post-stroke rats to elucidate whether it exerts anti-inflammatory effects that may explain its ability to alleviate the elevated BT (fever) observed in these animals. As seen in Figure 3, PGE2 levels were significantly higher in vehicle-treated MCAO-operated rats as compared to sham-operated rats (Figure 3A–C). Importantly, MEDS-23 treatment significantly decreased HT and HC PGE2 levels in MCAO-operated rats (Figure 3A,B, respectively). Furthermore, in general, the levels of IL-6 and TNF-α did not differ significantly between vehicle-treated sham-operated and MCAO-operated rats in the tested brain regions (Figure 3D–I). In contrast to our assumption, MEDS-23 treatment significantly increased IL-6 and TNF-α levels in HT of sham-operated rats (Figure 3D,G, respectively) whereas it did not significantly alter their levels in MCAO-operated rats (Figure 3D–I). As was seen, MEDS-23 treatment was generally not associated with a prominent effect on cytokine levels in post-stroke animals.

#### 3.2.4. Effects of MEDS-23 Treatment on NS of Post-Stroke Rats

**Figure 4:**
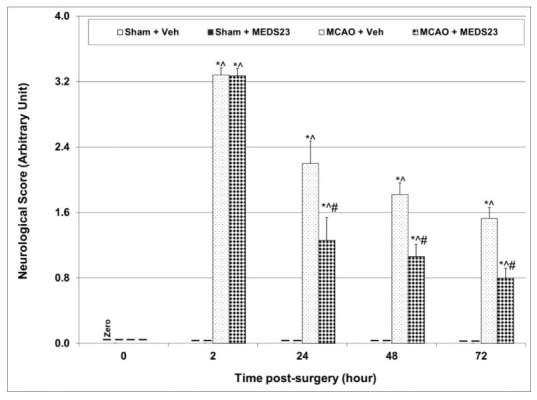
Effects of MEDS-23 treatment on NS of post-stroke rats. Sham- and MCAO-operated rats were treated with vehicle (DMSO 0.1 mL/rat) or MEDS-23 10 mg/kg for a total of 15 days through a single daily injection (i.p.). NS was assessed as described in “Materials and Methods” before and at 2, 24, 48 and 72 h post-surgery. The figure represents the combined results of all four *Efficacy Experiments*. Each point represents mean ± SEM of a different number of rats in each group at the various time-points of the experiment as shown in Table S5. Using multiple *t*-test—* *p* < 0.05 vs. Sham + Vehicle, ^ *p* < 0.05 vs. Sham + MEDS-23, # *p* < 0.05 vs. MCAO + Vehicle—at the same time-point. MCAO—middle cerebral artery occlusion; Veh—vehicle.

Figure 4 presents the mean value of the assigned NS to each group during the first three days of the *Efficacy Experiments*. At baseline (before surgery), all rats had a NS of “zero”, meaning that none of them had any visible neurological deficits. Subsequently, at 2, 24, 48 and 72 h post-surgery, all sham-operated rats maintained an NS of zero, irrespective of the treatment they received. In contrast, NS was significantly higher in all MCAO-operated rats at the same time-points; however, this increase was significantly attenuated by MEDS-23 treatment (Figure 4).

#### 3.2.5. Effects of MEDS-23 Treatment on Mortality of Post-Stroke Rats

**Figure 5:**
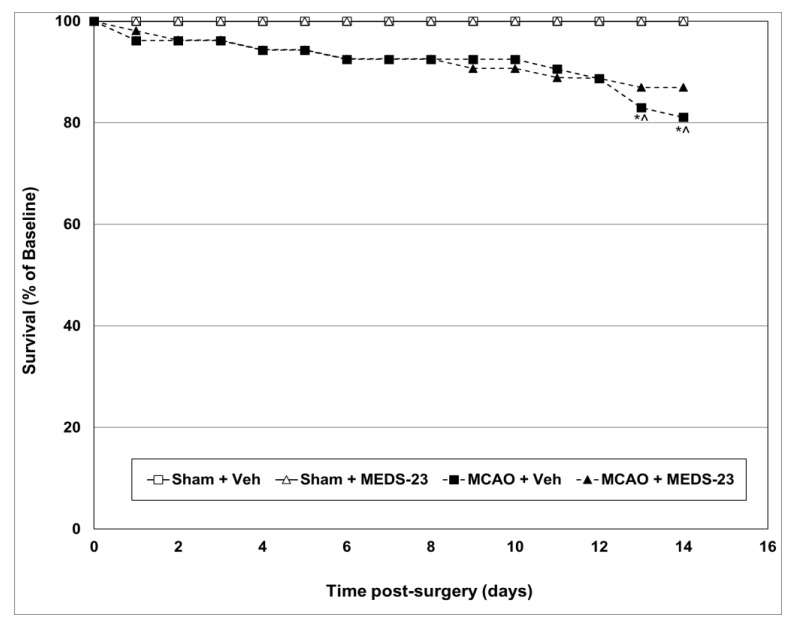
Effects of MEDS-23 treatment on mortality of post-stroke rats. Sham- and MCAO-operated rats were treated with vehicle (DMSO 0.1 mL/rat) or MEDS-23 10 mg/kg for a total of 15 days through a single daily injection (i.p.). Rat mortality was monitored for 14 days post-surgery. The figure represents the combined results of all four *Efficacy Experiments*. Survival rates at the various time-points of the experiment are presented as percentage of baseline survival which is expressed as 100%. The sample size in each group at baseline was as follows: Sham + Vehicle = 30, Sham + MEDS-23 = 31, MCAO + Vehicle = 53, MCAO + MEDS-23 = 54. An independent samples log-rank (Mantel-Cox) test revealed significant survival estimates between the groups (*p* = 0.028). Fisher-exact test was used to compare the difference between particular groups—* *p*< 0.05 vs. Sham + Vehicle, ^ *p* < 0.05 vs. Sham + MEDS-23—at the same time-point. MCAO—middle cerebral artery occlusion, Veh—vehicle.

Figure 5 presents the combined results of the survival rates of the groups during the course of the efficacy experiments. As seen, all sham-operated rats survived the 14 days of follow-up. In contrast, in the two MCAO-operated groups, mortality was already observed on the first post-operative day. At the 14th day of follow-up, the survival rates were 81% in the MCAO + vehicle group and 87% in the MCAO + MEDS-23 group. An independent samples log-rank (Mantel-Cox) test revealed significant (*p* = 0.028) survival estimates between all groups. A Fisher’s-exact test for comparisons of mortality rate between particular groups on specific days of the experiment revealed a significant difference between MCAO + vehicle group to the sham-operated groups on days 13 and 14 (*p* = 0.027). On the other hand, the mortality rate of the MCAO + MEDS-23 group did not differ significantly (*p* = 0.09) from that of the sham-operated groups. There was no significant difference between the two MCAO-operated groups during all the experiment days (*p* = 0.6 for the difference between the groups on day 14) (Figure 5).

#### 3.2.6. Effects of MEDS-23 Treatment on Sucrose Consumption in Post-Stroke Rats

The sucrose consumption test is used to assess depressive-like behavior. Decreased consumption of a sweetened solution resembles a state of anhedonia and represents depressive-like behavior. The amount of consumed sucrose solution may differ between different experiments because it is influenced by several factors (such as animals’ BW, ambient temperature and humidity, among others). We undertook significant efforts to ensure uniform conditions for the various experiments; nevertheless, some minor not noticeable changes in experimental conditions may still have existed. Therefore, Table 1 presents the results of a single efficacy experiment. As seen, at baseline there were no significant differences in sucrose consumption between the groups. On the other hand, on the 13th day of follow-up, a significantly lower sucrose consumption was observed in the MCAO-operated groups as compared to the sham-operated groups, suggestive of depressive-like behavior. MEDS-23 treatment did not significantly affect sucrose consumption in sham- as well as MCAO-operated rats (Table 1).

#### 3.2.7. Effects of MEDS-23 Treatment on Anxiety-Like Behavior in Post-Stroke Rats

The elevated plus-maze test is used to assess anxiety-like behavior. It measures the time animals spend in the closed arms of the maze (suggestive of anxious behavior) versus the time spent in the open arms (suggestive of non-anxious behavior). As seen in Table 2, on the 12th day of follow-up, the time spent in the closed arms was significantly lower in post-MCAO rats as compared to sham-operated rats. Accordingly, the time spent in the open arms was significantly higher in post-MCAO rats as compared to sham-operated rats, suggestive of non-anxious/manic-like behavior. Treatment with MEDS-23 did not significantly alter the tested measures in sham-operated rats as well as post-MCAO rats (Table 2).

## 4. Discussion

The major findings of the present study were that treatment with the potent NF-κB inhibitor MEDS-23 significantly attenuated the elevated BT (fever) and decreased the severity of neurological deficits in post-ischemic stroke rats. On the other hand, MEDS-23 did not significantly reduce post-stroke mortality nor did it prominently influence the levels of brain IL-6 and TNF-α in post-stroke rats.

A large body of data indicates that activation of NF-κB contributes to the pathophysiology of ischemic stroke and, therefore, inhibition of NF-κB has been suggested as a potential therapeutic strategy against post-stroke brain inflammation [32,33,34]. This was the basis for the use of MEDS-23—a potent NF-κB inhibitor [11]—in the MCAO rat model of ischemic stroke. We hypothesized that MEDS-23 will attenuate post-stroke morbidity and mortality due to its potential anti-inflammatory effects. We tested the effects of MEDS-23 on several adverse clinical outcomes that are prevalent in post-stroke patients including fever, neurological deficits, mortality, and depressive symptoms.

Several studies demonstrated that post-stroke fever negatively affects the prognosis of patients after ischemic stroke [35,36,37]. In the present study, we found that post-MCAO rats had a significantly elevated BT for three days post-surgery (Figure 2). This is consistent with the results of previous studies in post-stroke rats [21,22]. Treatment with MEDS-23 significantly attenuated the elevated BT in post-MCAO rats (Figure 2), suggestive of a potent antipyretic effect of this NF-κB inhibitor. The mechanism underlying post-stroke fever is not well understood. Several hypotheses have been suggested to explain the pathogenesis of post-stroke fever [37,38,39]. One of the proteins that is thought to play a crucial role in the regulation of BT during pathological conditions is PGE2. The thermoregulation zone in the brain is located in the HT. Conditions such as ischemic injury [40] and inflammatory insults [41] have been shown to increase nuclear NF-κB levels and cyclooxygenase (COX)-2 (which produces PGE2 mainly during pathological conditions) expression in the HT. Thus, it is important to assess HT PGE2 levels in order to understand whether it is involved in the mechanism of post-stroke fever. A previous study from our laboratory demonstrated that acute treatment with the mood stabilizer lithium did not attenuate post-stroke fever in rats despite causing a prominent reduction in HT PGE2 levels [21]. This suggests that an increase in HT PGE2 levels is not the sole factor that may lead to post-stroke fever. Consistently, aspirin, a drug that inhibits PGE2 production, did not reduce post-MCAO fever in rats [42]. In contrast to these findings, we recently showed that chronic treatment with mofezolac—a selective COX-1 inhibitor—significantly decreased post-MCAO fever in rats, which was accompanied by a significant reduction in PGE2 levels in the HT (and also in the FC and HC) [22]. Similarly, in the present study, the prominent reduction of post-MCAO fever in MEDS-23-treated rats was accompanied by a significant decrease in PGE2 levels in the HT and HC (Figure 3A,B). Therefore, it is possible that an elevation in HC PGE2 levels also contributes to the development of post-stroke fever. Additionally, PGE2-independent mechanisms should not be ruled out; it is possible that inhibition by MEDS-23 of other down-stream targets of NF-κB may have contributed to its antipyretic effect in post-MCAO rats.

We tested the effects of MEDS-23 on levels of the inflammatory mediators IL-6 and TNF-α in brains of post-stroke rats to elucidate a possible association between its antipyretic effect and its correlation with neuro-inflammation. Surprisingly, IL-6 levels were not significantly increased in HT and FC of MCAO-operated rats as compared to sham-operated rats (Figure 3). Similarly, TNF-α levels were not significantly increased in HT, HC and FC of MCAO-operated rats (Figure 3). These findings are similar to those of our previous study [22] in which IL-6 and TNF-α levels were not found elevated in the brains of post-stroke rats at 14 days post-MCAO. This may be related to the design of the study and the fact that the brains of experimental rats were extracted at 14 days after the induction of stroke, when the magnitude of the inflammatory process in the brain has already declined. This design was selected primarily in order to evaluate the effects of *chronic* MEDS-23 treatment on adverse clinical outcomes of post-stroke rats. MEDS-23 treatment did not decrease brain levels of IL-6 (Figure 3D–F) and TNF-α (Figure 3G–I) in MCAO-operated rats. MEDS-23 significantly increased HT IL-6 (Figure 3D) and TNF-α (Figure 3G) levels in sham-operated rats. We speculated that MEDS-23, as a potent inhibitor of NF-κB [11], would reduce the levels of these pro-inflammatory cytokines because under most experimental conditions activation of NF-κB leads to increased production of pro-inflammatory cytokines. However, consistent with these unexpected results, a preliminary study in our lab demonstrated that JSH-23—a selective inhibitor of NF-κB [43]—also did not alter brain IL-6 and TNF-α levels in MCAO-operated rats after chronic treatment [Hijaze, Nassar, Boyko and Azab—personal communication]. Moreover, it cannot be ruled out that the absence of a robust inhibitory effect of MEDS-23 on IL-6 and TNF-α production derives from the lack of a prominent increase in their levels in post-MCAO rats at the examined time-point.

Among the most severe adverse clinical outcomes occurring after ischemic stroke are the neurological deficits and mortality seen in these patients [44,45,46]. As seen in Figure 5, all rats that underwent MCAO surgery had prominent visible neurological deficits at nearly two hours post-surgery. The severity of neurological deficits gradually decreased during the three days of follow-up. Treatment with MEDS-23 significantly decreased the NS at 24, 48 and 72 h post-surgery in MCAO-operated rats (Figure 4). Furthermore, we found that the cumulative mortality in vehicle-treated-MCAO-operated rats was significantly higher (*p* = 0.027) than that of sham-operated rats at certain days of the follow-up (Figure 5). On the other hand, the cumulative mortality in the MEDS-23-treated-MCAO-operated rats was not significantly higher (*p* = 0.09) than that of sham-operated rats, suggestive of a possible protective effect of MEDS-23 against post-stroke mortality. Of note, the difference between vehicle-treated and MEDS-23-treated MCAO-operated rats was not significant (*p* = 0.6). Together, these findings indicate that early administration of a NF-κB inhibitor (the first injection of MEDS-23 was four hours after the induction of stroke) and its subsequent continuation is capable of mitigating the severity of neurological deficits in post-stroke rats. Consistent with previous results [32,47,48], this finding highlights the potential of NF-κB inhibition as a therapeutic strategy for reducing post-stroke disabilities.

Depression is another prominent adverse clinical outcome in post-stroke patients—nearly one third of stroke patients develop depression [49]. Depression in post-stroke patients is associated with slow recovery and increased mortality [39]. Anxiety is a common feature among patients with depressive disorders, including post-stroke (depressed) patients. A large body of data has linked NF-κB to the pathophysiology and treatment of mood disorders [50,51,52,53]. For instance, it was found that NF-κB levels are elevated in patients with mood disorders [50,53] and that psychotropic drugs alter the function of the NF-κB machinery [51]. Therefore, in the present study, we examined the effects of MEDS-23 treatment on the behavioral phenotype of post-MCAO rats in two models used for the assessment of depressive-like and anxiety-like behaviors: the sucrose consumption test and the elevated plus-maze test, respectively. We found that sucrose consumption was significantly lower in MCAO-operated rats as compared to sham-operated rats, suggestive of depressive-like behavior (Table 1). MEDS-23 treatment did not influence the diminished sucrose consumption in MCAO-operated rats (Table 1). Moreover, in the elevated plus-maze test, we found that MCAO-operated rats spent significantly more time in the open arms of the maze (as compared to sham-operated rats), suggestive of non-anxious/manic-like behavior (Table 2). Treatment with MEDS-23 did not significantly alter the time spent in the open arms neither in sham-operated nor in MCAO-operated rats (Table 2). Consistent with our results, a previous study similarly demonstrated a significantly increased time spent in the open arms in post-MCAO rats as compared to sham-operated rats [54]. We expected MCAO-operated rats to spend less time in the open arms of the maze, which is consistent with anxious-like behavior. Naturally, rodents hide in dark places during light time to avoid the possibility of being seen by enemies. Thus, the choice to stay in the closed arms of the maze represents a normal behavior when the proportion of the time spent in these arms is similar or not significantly higher than that in control animals. On the other hand, going to the open arms is usually recognized as a non-anxious behavior. If the time spent in the open arms is dramatically higher than that seen in control animals, it may be interpreted as a risk-taking/manic behavior because it suggests that the animals are not afraid to take the risk of being seen by enemies. We speculate that the increased time spent in the open arms in MCAO-operated rats may derive from stroke-induced damage to cognitive function disrupting their judgment and decision-making process to avoid possible enemies. Collectively, these results suggest that under the current experimental conditions it is not possible to unambiguously associate an improvement in behavior with inhibition of NF-κB by MEDS-23 in post-stroke rats.

Study limitations. The present study has some limitations: (1) The behavioral models used in this study do not fulfill all validity criteria for depression and anxiety in humans. The paucity of appropriate validated models led us to choose those acceptable in the field and feasible for our study design; (2) The inclusion of only male rats is another limitation of the study. Ischemic stroke is prevalent in women no less than it is in men [55]. Moreover, women were reported to suffer poorer stroke outcomes including higher rates of complications, prolonged hospital stays, and in-hospital death [55]. The choice of including only male rats was made because in preliminary experiments we observed that post-surgical recovery time in female rats was significantly longer than in male rats. The inclusion of female rats would have complicated the design of the study; (3) Modification of the study design may lead to better therapeutic effects in future studies. For example, it is possible that a longer duration of MEDS-23 treatment would enhance its therapeutic efficacy. This is supported by our observations that in some of the tested parameters (e.g., NS and mortality) we identified a better improvement in MEDS-23-treated MCAO-operated rats during the last few days of follow-up. Additionally, it is possible that administering an initial loading-dose of MEDS-23 (e.g., 50 mg/kg instead of 10 mg/kg) immediately after the induction of stroke may also result in better efficacy. An initial high dose of MEDS-23 may better alleviate the prominent acute inflammatory response that occurs in the brain immediately after the acute ischemia. Furthermore, future studies may benefit from including a behavioral test which assesses post-stroke cognitive impairment, as vascular cognitive impairment is a significant adverse event after ischemic stroke.

## 5. Conclusions

This study demonstrated that chronic treatment with the potent NF-κB inhibitor MEDS-23 reduced the severity of some post-stroke adverse outcomes such as fever and neurological deficits. On the other hand, it did not significantly affect post-stroke mortality. Further research is necessary to elucidate the role of NF-κB in the pathophysiology and treatment of post-stroke animals.

## Figures and Tables

**Table 1:** Effects of MEDS-23 treatment on sucrose consumption in post-stroke rats.

	Baseline, Mean ± SEM	After 13 Days, Mean ± SEM
Sham + Vehicle (*n* = 10)	1.62 ± 0.18	1.73 ± 0.30
Sham + MEDS-23 (*n* = 10)	1.97 ± 0.38	1.80 ± 0.18
MCAO + Vehicle (*n* = 16)	1.92 ± 0.56	0.94 ± 0.43 ^#,^*^,^^
MCAO + MEDS-23 (*n* = 16)	1.77 ± 0.33	1.08 ± 0.23 ^#,^*^,^^

Sham- and MCAO-operated rats were treated with vehicle (DMSO 0.1 mL/rat) or MEDS-23 10 mg/kg for a total of 15 days through a single daily injection (i.p.). Twenty-four hour sucrose consumption was evaluated as described in “Materials and Methods” before (baseline) and at 13 days post-surgery. The table represents the results of a single *Efficacy Experiment* (very similar results were obtained in other experiments). Values express gram sucrose per kilogram body weight. Using paired samples *t*-test—^#^ *p* < 0.05 vs. the same group at baseline. Using independent *t*-test—* *p* < 0.05 vs. Sham + Vehicle, ^^^ *p* < 0.05 vs. Sham + MEDS-23 at the same time. MCAO—middle cerebral artery occlusion.

**Table 2:** Effects of MEDS-23 treatment on anxiety-like behavior in post-stroke rats.

Group	Sham + Vehicle (*n* = 9)	Sham + MEDS23 (*n* = 8)	MCAO + Vehicle (*n* = 14)	MCAO + MEDS23 (*n* = 14)
Time in closed arm (sec); mean ± SEM	214.2 ± 11.2	213.8 ± 10.9	178.1 ± 15.6 *^,^^	165.3 ± 13.5 *^,^^
Time in open arm (sec); mean ± SEM	85.8 ± 11.2	86.2 ± 10.9	121.9 ± 15.6 *^,^^	134.7 ± 13.5 *^,^^

Sham- and MCAO-operated rats were treated with vehicle (DMSO 0.1 mL/rat) or MEDS-23 10 mg/kg for a total of 15 days through a single daily injection (i.p.). Five minutes sessions of the EPMT were conducted as described in “Materials and Methods” on the 12th day post-surgery. The table represents the results of a single *Efficacy Experiment* (very similar results were obtained in other experiments). Using independent *t*-test—* *p* < 0.05 vs. Sham + Vehicle, ^ *p* < 0.05 vs. Sham + MEDS-23 at the same time. MCAO—middle cerebral artery occlusion.

## Data Availability

The primary data used to support the findings of this study are available from the corresponding author upon reasonable request.

## Supplementary Materials

The following supporting information can be downloaded at: https://www.mdpi.com/article/10.3390/brainsci12010035/s1. Section S1: Details about the chemistry of MEDS-23 preparation in multi-gram scale. Section S2: Tables and figures about the toxicity experiment. Section S3: Table S5 presents the sample size of the treatment groups throughout the efficacy experiments.
