# Supplementary material for: A New NF-κB Inhibitor, MEDS-23, Reduces the Severity of Adverse Post-Ischemic Stroke Outcomes in Rats
Source: Brain Sci. 2021 Dec 28;12(1):35. doi: 10.3390/brainsci12010035 (PMC8773493; doi:10.3390/brainsci12010035)
Supplement: Supplementary file 1 [file brainsci-12-00035-s001.zip › brainsci-1524733-supplementary.pdf]

To: *Brain Sciences*

Supplementary Material

A new NF-κB inhibitor, MEDS-23, reduces the severity of adverse post-ischemic stroke outcomes in rats

Elina Rubin[1,#], Agnese Chiara Pippione[2,#], Matthew Boyko[3], Giacomo Einaudi[4], Stefano Sainas[2], Massimo Collino[5], Carlo Cifani[4], Marco Lolli[2], Naim Abu-Freha[6], Jacob Kaplanski[1], Donatella Boschi[2] and Abed N. Azab[1,7]*]

[1]Department of Clinical Biochemistry and Pharmacology, Ben-Gurion University of the Negev, P.O.B 653, Beer-Sheva 8410501, Israel; [2] Department of Drug Science and Technology, University of Turin, 10125, Italy; [3] Department of Anesthesiology and Critical Care, Soroka University Medical Center, Ben-Gurion University of the Negev, Beer-Sheva, Israel; [4] Pharmacology Unit, School of Pharmacy, University of Camerino, 62032, Italy; [5] Department of Neuroscience "Rita Levi Montalcini", University of Turin, 10125, Italy; [6] Institute of Gastroenterology and Hepatology, Soroka University Medical Center, Ben-Gurion University of the Negev, Beer-Sheva, Israel; [7] Department of Nursing, Ben-Gurion University of the Negev, P.O.B 653, Beer-Sheva 8410501, Israel.

[#]These authors contributed equally.

*Correspondence should be addressed to: Dr. Abed N. Azab; azab@bgu.ac.il

Table of contents:

S1. Chemistry	Pages S2-S5
S2. Toxicity Experiment	Pages S5-9
S3. Efficacy Experiments	Page S9

S1. Chemistry

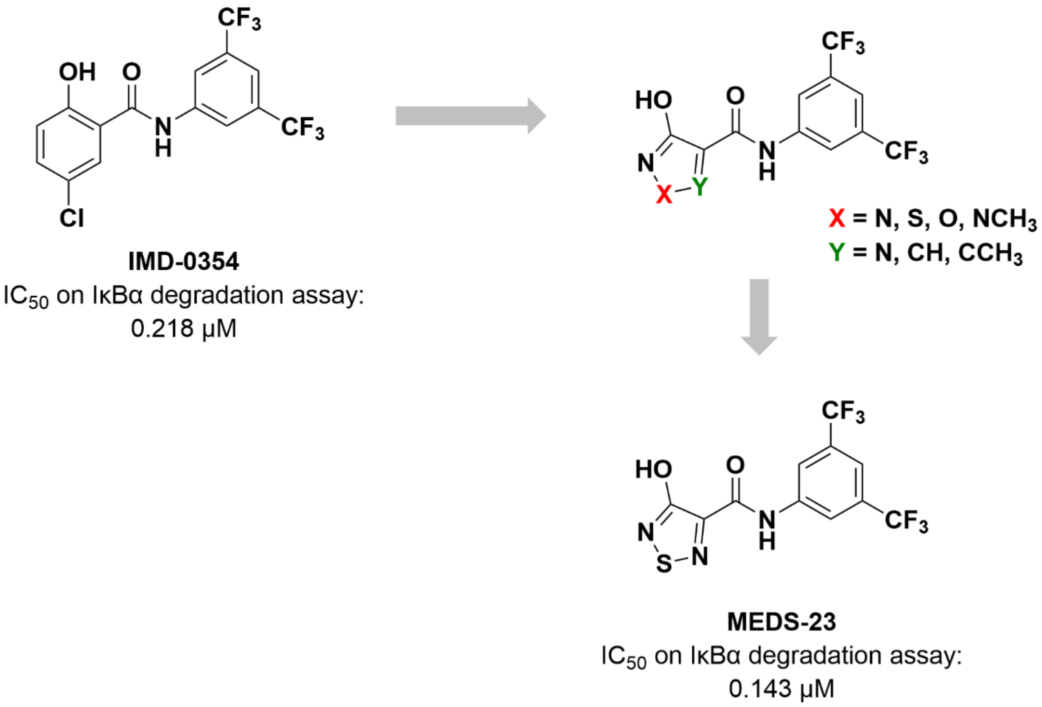

Figure S1: NF-κB inhibitors IMD-0354 and MEDS-23, the last is the object of the present study.

S1.1. Synthesis of MEDS-23.

Several grams of MEDS-23 were consumed for *in-vivo* experiments. Such high amounts of the compound could not be synthesised with the protocol originally developed, therefore a new synthetic scheme was developed to scale up the procedure (Scheme S1).

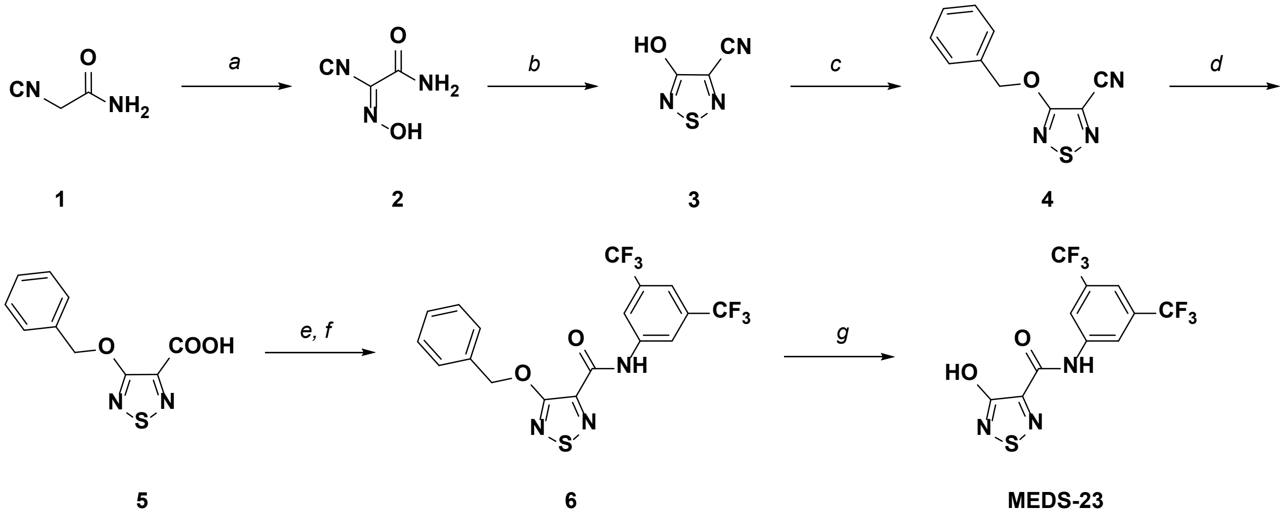

Scheme S1: Reagent and conditions: a) $NaNO_2$, HCl, H_2O; b) S_2Cl_2, acetonitrile, reflux; c) benzyl bromide, K_2CO_3, dimethylformamide, 60° C; d) 2M NaOH, MeOH, reflux; e) $(COCl)_2$, dimethylformamide, THF; f) 3,5-bis(trifluoromethyl)aniline, pyridine, THF; g) trifluoacetic acid, thioanisole. 80° C.

The synthetic scheme designed for MEDS-23 started from cyanoacetamide **1**, that was transformed into its hydroxyimino derivative **2** and then closed to hydroxythiadiazole **3** following already described procedures. The protection of the hydroxyl group of **3** with benzyl group was necessary (compound **4**). Benzyl group is a suitable protective group for the hydroxylated azoles, but not for the hydroxylated thiadiazole for which the deprotection in standard condition (catalytic hydrogenation or TFA) was challenging. In fact, the presence of sulfur atom poisoned the Pd/C catalyst, while benzyl cation originating by deprotection with TFA reacted with annular nitrogen atom giving an alkylated byproduct and affecting the yield. For this reason, we previously described the obtainment of thiadiazole amides without protecting hydroxy function of 4-hydroxy-1,2,5-thiadiazole-3-carboxylic acid (see Pippione *et al.*, *MedChemComm,* 2017, 8, 1850-1855). Here instead, we use benzyl as protective group and removed it in the final passage using TFA and the benzyl scavenger thioanisole, avoiding formation of byproducts.

Compound **4** was subjected to hydrolysis of the nitrile moiety, obtained in basic environment in refluxing methanol (compound **5**). Carboxylic acid **5** was transformed into its acyl chloride and then coupled with bis(trifluoromethyl)aniline, obtaining the protected thiadiazole amide **5.** Subsequent treatment of **5** with trifluoroacetic acid and benzyl scavenger thioanisole under moderate heating gave the final compound MEDS-23.

S1.2. General methods. All chemical reagents were obtained from commercial sources (Sigma Aldrich, Alfa Aesar), and used without further purification. Thin-layer chromatography (TLC), was performed to monitor the reaction processes. Analytical grade solvents (acetonitrile, diisopropyl ether, diethyl ether, dichloromethane [DCM], dimethylformamide [DMF], ethanol 99.8 % v/v, ethyl acetate, methanol [MeOH], petroleum ether b.p. 40 - 60 °C [petroleum ether]), were used without

further purification. When required, solvents were dried on 4 Å molecular sieves. Tetrahydrofuran (THF), was distilled from Na and benzophenone under N_2 immediately prior to use. Thin layer chromatography (TLC), was carried out on silica gel on 5 x 20 cm plates with a 0.25 mm layer thickness. Anhydrous $MgSO_4$ was used as a drying agent for the organic phases. Compound purification was achieved using flash column chromatography on silica gel (Merck Kieselgel 60, 230-400 mesh ASTM), and the eluents indicated. Compounds synthesized in our laboratory generally varied between 90 % and 99 % purity. Biological experiments were carried out on compounds (MEDS-23) with a purity of at least 95%. Purity was checked using HPLC analyses, performed on a UHPLC chromatographic system (Perkin Elmer, Flexar). The analytical column was a UHPLC Acquity CSH Fluoro-Phenyl system (2.1 x 100 mm, 1.7 μm particle size) (Waters). Compounds were dissolved in MeOH and injected through a 20 μl loop. The mobile phase consisted of acetonitrile/water with 0.1 % trifluoroacetic acid; two mobile phase gradient profiles were used to assay the purity of each compound. UHPLC retention times were obtained at flow rates of 0.5 mL/min and the column effluent was monitored at 215 and 254 nm, referenced against a 360 nm wavelength. Melting points (m.p.), were measured on a capillary apparatus (Büchi 540). Final m.p. determination was achieved by placing the sample at a temperature that was 10° C below the m.p. and applying a heating rate of 1° C min^{-1}. ^1H- and ^{13}C-NMR spectra were performed on a Bruker Avance 300 instrument or Jeol Resonance ECZ600R. MS spectra were performed on Waters Micromass ZQ equipped with ESCi source for electrospray ionization mass spectra (ESI).

S1.3. 2-cyano-2-(hydroxyimino)acetamide (2). 20.00 grams (0.238 mol) of cyanoacetamide **1** were slowly added to concentrated HCl at 0° C. To this suspension, a solution of $NaNO_2$ (19.70 grams in 70 ml of water) was added, maintaining the temperature between 0° and 5° C degrees, until brown fumes stop to develop. The resulting solid is filtered and washed with water, obtaining compound **2**. Yield 68 %. White solid, m.p. 175.0-177.4° C from diethyl ether.

S1.4. 4-hydroxy-1,2,5-thiadiazole-3-carbonitrile (3). Disulfur dichloride (7.00 mL, 0.088 mol) in acetonitrile was added to a suspension of **2** (6.00 grams, 0.053 mol) in acetonitrile, heating the mixture at 60° C and removing the vapors into a trap. The mixture was then heated at reflux for 3 hours, then cooled to room temperature and quenched in iced water (200 mL). The resulting suspension is filtered, and the filtrate is extracted with dichloromethane (3 x 100 mL), the collected organic layer was washed with brine, dried on Na_2SO_4 and the solvent was evaporated to give **3** as an orange solid. Yield 70 %. m.p. 150.1-153.5° C.

S1.5. 4-(benzyloxy)-1,2,5-thiadiazole-3-carbonitrile (4). Obtained from **4** as described by Sainas *et al.*, *Eur J Med Chem*, 2017, 129, 287-302.

S1.6. 4-(benzyloxy)-1,2,5-thiadiazole-3-carboxylic acid (5). Obtained from **5** as described by Sainas *et al.*, *Eur J Med Chem*, 2017, 129, 287-302.

S1.7. 4-(benzyloxy)-N-[3,5-bis(trifluoromethyl)phenyl]-1,2,5-thiadiazole-3-carboxamide (6). Dry DMF (0.25 mL) and oxalyl chloride (25.4 mmol, 2.15 mL) were added to a cooled (0° C) solution of **4** (10.6 mmol, 2.50 g) in dry THF (60 mL). The resulting solution was stirred for 2 h at room temperature under nitrogen atmosphere. Then the solvent was evaporated under reduced pressure and the residue was dissolved in dry THF (this step was repeated three times). The resulting 4-(benzyloxy)-1,2,5-thiadiazole-3-carbonyl chloride was dissolved in dry THF (30 mL) under nitrogen atmosphere and added with a solution of 3,5-bis(trifluoromethyl)aniline (10.6 mmol, 1.65 mL) and dry pyridine (31.8 mmol, 2.57 mL) in dry THF. The mixture was stirred overnight at room temperature. The suspension was quenched with 2M HCl (120 mL); a white solid precipitated. The solid was filtered and washed with water and hexane, then purified by flash chromatography (eluent: petroleum ether/ethyl acetate from 85/15 to 0/100 v/v) to obtain a white solid (m.p. 176.1-178.3° C). Yield 61 %. ^{1}H-NMR (600 MHz, $CDCl_3$) δ 5.58 (s, 2H), 7.37-7.46 (m, 3H), 7.53 (d, J = 7.0 Hz, 2H), 7.63 (s, 1H), 8.11 (s, 2H), 9.19 (s, 1H). ^{13}C-NMR (151 MHz, $CDCl_3$) δ 73.6, 118.2, 119.6, 123.1 (q,

J = 272.9 Hz), 128.5, 128.9, 129.1, 132.7 (q, J =33.8 Hz), 134.9, 138.8, 140.5, 156.2, 163.9. MS (ESI): (m/z) 448 [M+1].

S1.8. 4-Hydroxy-N-[3,5-bis(trifluoromethyl)phenyl]-1,2,5-thiadiazole-3-carboxamide (MEDS-23). 2.00 grams (4.5 mmol) of **5** were dissolved in trifluoroacetic acid (10 mL) and thioanisole (3.15 mL, 27 mmol) and heated at 80°C for 4 hours. After cooling, the suspension was quenched in water (about 150 mL), resulting in an emulsion that was extracted with EtOAc (3x100 mL). The organic phases were washed with brine, dried with Na_2SO_4 and concentrated on reduced pressure. The residue (about 20 mL) was left at room temperature for 1 night and a white needle-shaped solid precipitated. The solid was filtered, well washed with hexane (to remove thioanisole) and water (to remove TFA) to give the desired compound. White solid (m.p. 208.0 – 209.6 °C from hexane). Yield 72 %. ^{1} H-NMR (300 MHz, CD_3OD): δ 7.73 (1H, s, aromatic proton), 8.45 (2H, s, aromatic protons); ^{13}C-NMR (75 MHz CD_3OD): δ 118.5 (q, 3 J_{CF} = 3.8 Hz), 121.4 (q, 3 J_{CF} = 4.1 Hz), 124.7 (q, 1 J_{CF} = 271.6 Hz), 133.3 (q, 2 J_{CF} = 33.4 Hz), 140.5, 141.1, 160.7, 166.0. MS (ESI): (m/z) 357 [M+1].

S2. Toxicity Experiment.

S2.1. Body weight. At baseline, each group consisted of six rats and the average BW was identical in all treatment groups (mean ± SEM = ~ 246 ± 2 g). As seen in Figure S2, BW is presented as percentage of baseline, calculated according to the following formula: [BW of a certain day/BW of day “zero”] * 100. BW was similar in the first four groups (control, MEDS-23 1, 5 or 10 mg/kg) and no significant differences were observed between them at any time-point. Nevertheless, it is worth emphasizing that the MEDS-23 5 and 10 mg/kg doses resulted in a slight but non-significant decrease in BW of approximately 2-3%. In contrast, in the MEDS-23 50 mg/kg group, from experiment day 2, BW was significantly lower than that of the control group, suggestive of a toxic effect. In the 100 mg/kg group, BW was also significantly lower on experiment day 2, which was

accompanied by the death of two rats. After experiment day 3, all animals in the 100 mg/kg group died, suggestive of severe toxicity (Figure S2).

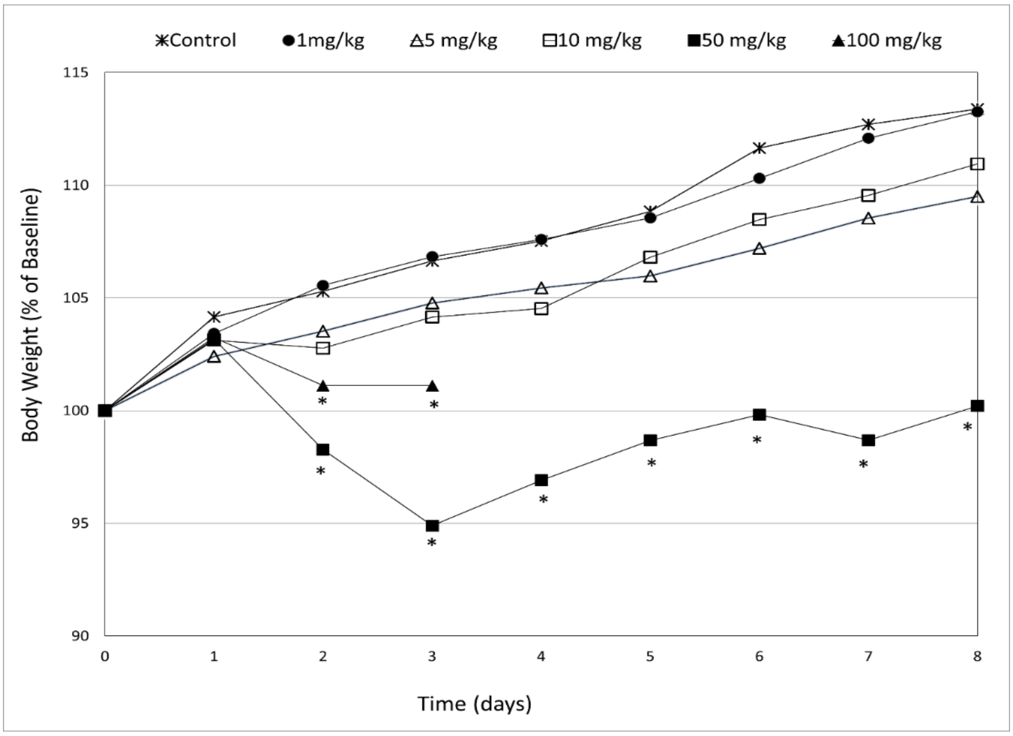

Figure S2: Effects of MEDS-23 on rats' BW. Naïve rats were treated with vehicle (DMSO 0.2 ml/rat) or MEDS-23 1, 5, 10, 50, or 100 mg/kg for 7 days through a single daily ip injection. BW was measured every day before MEDS-23 or DMSO injection. Each point represents mean ± SEM of six rats in each group (except 100 mg/kg group). Using dependent samples t-test – * $P < 0.05$ vs. control - at the same time point.

S2.2. Survival. Table S1 presents the sample size and percentage of the survival rate in each group. As seen, in all groups but the MEDS-23 100 mg/kg, all six animals in each group survived (100% survival). In contrast, in the MEDS-23 100 mg/kg, all six animals died after the third day of the experiment (0% survival) (Table S1).

Table S1. Effects of MEDS-23 on rats' survival.

	Day 0	Day 1	Day 2	Day 3	Day 4	Day 5	Day 6	Day 7
Control, n (%)	6 (100)	6 (100)	6 (100)	6 (100)	6 (100)	6 (100)	6 (100)	6 (100)
1 mg/kg, n (%)	6 (100)	6 (100)	6 (100)	6 (100)	6 (100)	6 (100)	6 (100)	6 (100)
5 mg/kg, n (%)	6 (100)	6 (100)	6 (100)	6 (100)	6 (100)	6 (100)	6 (100)	6 (100)
10 mg/kg, n (%)	6 (100)	6 (100)	6 (100)	6 (100)	6 (100)	6 (100)	6 (100)	6 (100)
50 mg/kg, n (%)	6 (100)	6 (100)	6 (100)	6 (100)	6 (100)	6 (100)	6 (100)	6 (100)
100 mg/kg, n (%)	6 (100)	3 (50)	3 (50)	2 (33)	0 (0)	0 (0)	0 (0)	0 (0)

Naïve rats were treated with vehicle or MEDS-23 1, 5, 10, 50, or 100 mg/kg for seven days through a single daily ip injection and survival was monitored. Shown are the number and percentage (parenthesis) of surviving animals at each time-point. Using Fisher Exact test – no significant differences between the groups.

S2.3. Kidney function parameters. Table S2 presents values of creatinine and urea (considered basic determinants of renal function) in control and MEDS-23-treated at the end of the *Toxicity Experiment*. As seen, creatinine and urea levels did not significantly differ between the control group and those of rats treated with MEDS-23 1, 5 or 10 mg/kg. On the other hand, creatinine levels were significantly higher in the 50 mg/kg group as compared to control (Table S2), suggestive of a possible renal toxicity of this dose. Of note, urea levels were also non-significantly increased in this group.

Table S2. Effects of MEDS-23 on creatinine and urea levels.

Group	Control (n=6)	1 mg/kg (n=6)	5 mg/kg (n=6)	10 mg/kg (n=6)	50 mg/kg (n=6)
Creatinine (mg/dL)	0.21 ± 0.01	0.25 ± 0.02	0.20 ± 0.01	0.23 ± 0.02	0.27 ± 0.02 *
Urea (mg/dL)	53.0 ± 2.25	57.9 ± 2.31	50.8 ± 2.96	50.4 ± 3.36	67.2 ± 8.18

Naïve rats were treated with vehicle or MEDS-23 1, 5, 10, or 50 mg/kg for 7 days through a single daily ip injection. Then, rats were euthanized and blood was collected for determination of renal function parameters. Results are presented as mean ± SEM of 6 rats per group. Using dependent samples t-test – * $P < 0.05$ vs. control for the same parameter.

S2.4. Hematological parameters. Table S3 presents hematological parameters in control and MEDS-23-treated rats at the end of the *Toxicity* experiment. As seen, the blood cell count indicates that all MEDS-23-treated rats had hematological parameters that were similar to those of the control group. This suggests that MEDS-23 (up to 50 mg/kg) had no hematological toxicity.

Table S3. Effects of MEDS-23 on blood cell count.

Group	Control (n=6)	1 mg/kg (n=6)	5 mg/kg (n=6)	10 mg/kg (n=6)	50 mg/kg (n=6)
HB (g/dl); mean ± SEM	15.0 ± 0.2	15.1 ± 0.18	14.3 ± 0.43	15.1 ± 0.24	13.2 ± 1.7
RBC (*10^6/µL); mean ± SEM	7.9 ± 0.2	7.8 ± 0.1	7.5 ± 0.2	7.77 ± 0.2	6.8 ± 0.9
WBC (*10^3/µL); mean ± SEM	9.4 ± 0.6	9.8 ± 0.7	10.1 ± 1.5	10.2 ± 0.6	12 ± 1.6
PLT (*10^3/µL); mean ± SEM	907 ± 104	882 ± 120	880 ± 104	735 ± 98	768 ± 140

Naïve rats were treated with vehicle or MEDS-23 1, 5, 10, and 50 mg/kg for 7 days through a single daily ip injection. Then, rats were euthanized and blood was collected for determination of blood cell count. Results are presented as mean ± SEM of 6 rats per group. Abbreviations: HB - hemoglobin, RBC - red blood cells, WBC - white blood cells, PLT - platelets.

S2.5. Gastric mucosa integrity. We decided to assess gastric mucosal integrity as another toxicity measure for evaluating the safety of the new compound. Table 4 shows the prevalence of different adverse effects that were inspected in the stomach of rats treated with MEDS-23, including gastritis and atrophy. Rats from control, 1 mg/kg and 5 mg/kg had the same presence of gastritis. Gastritis was prevalent in rats treated with 10 mg/kg and 50 mg/kg of MEDS-23. Moderate to severe atrophy was found in two rats in the 50 mg/kg group.

Table S4. Effects of MEDS-23 on gastric mucosal integrity/structure.

	Control (n=6)	1 mg/kg (n=6)	5 mg/kg (n=6)	10 mg/kg (n=6)	50 mg/kg (n=6)
Normal, n (%)	5 (83)	5 (83)	4 (67)	1 (17)	0 (0)
Mild gastritis, n (%)	1 (17)	1 (17)	1 (17)	3 (50)	4 (66)
Moderate to severe gastritis, n (%)	0 (0)	0 (0)	1 (17)	2 (33)	2 (33)
Moderate to severe atrophy, n (%)	0 (0)	0 (0)	0 (0)	0 (0)	2 (33)

Naïve rats were treated with vehicle or MEDS-23 1, 5, 10 or 50 mg/kg for 7 days through a single daily ip injection. Then, rats were euthanized and stomachs were ousted and evaluated for the presence of macroscopic changes in gastric mucosal integrity/structure (no significant differences between the groups, Fisher Exact test).

S2.6. General health condition. Rats that were treated with MEDS-23 50 mg/kg seemed less socially active and less inclined to move compared to other groups after the third day of the experiment. Together with the other safety/toxicity findings (sections 3.1 and S2.1-2.5), these observations led us to exclude the 50 mg/kg dose (and of course the 100 mg/kg dose) for use in the *Efficacy Experiments*. Thus, we chose to use 10 mg/kg MEDS-23 in all *Efficacy Experiments*.

S3. Efficacy Experiments.

Table S5. Sample size of treatment groups throughout the efficacy experiments. MCAO - middle cerebral artery occlusion.

Group \ Day	0	1	2	3	4	5	6	7	8	9	10	11	12	13	14
Sham + Vehicle	30	30	30	30	30	30	30	30	30	30	30	30	30	30	30
Sham + MEDS-23	31	31	31	31	31	31	31	31	31	31	31	31	31	31	31
MCAO + Vehicle	53	51	51	51	50	50	49	49	49	49	49	48	47	44	43
MCAO + MEDS-23	54	53	52	52	51	51	50	50	50	49	49	48	48	47	47
